# Electron Beam Processing as A Promising Tool to Decontaminate Polymers Containing Brominated Flame Retardants

**DOI:** 10.3390/molecules28237753

**Published:** 2023-11-24

**Authors:** Rachida Khadidja Benmammar, Venkateswara Rao Mundlapati, Zohra Bouberka, Ana Barrera, Jean-Noël Staelens, Jean-François Tahon, Michael Ziskind, Yvain Carpentier, Cristian Focsa, Philippe Supiot, Corinne Foissac, Ulrich Maschke

**Affiliations:** 1 Unité Matériaux et Transformations (UMET), UMR 8207, CNRS, INRAE, Université de Lille, 59000 Lille, France; 2 Physique des Lasers Atomes et Molécules (PhlAM), UMR 8523, CNRS, Université de Lille, 59000 Lille, France; 3 Laboratoire Physico-Chimique des Matériaux, Catalyse et Environnement (LPMCE), Université des Sciences et de la Technologie Mohammed Boudiaf d’Oran (USTO-MB), Oran 31000, Algeria

**Keywords:** recycling, acrylonitrile-butadiene-styrene, polycarbonate, e-waste, electron-beam processing, brominated flame retardants

## Abstract

Electron Beam (EB) irradiation was utilized to decontaminate model systems of industrial polymers that contain a brominated flame retardant (BFR). Acrylonitrile-butadiene-styrene (ABS) and Polycarbonate (PC) are two types of polymers commonly found in Waste Electrical and Electronic Equipment (WEEE). In this study, these polymers were exposed to EB irradiation to degrade DecaBromoDiphenylEther (DBDE), one of the most toxic BFRs. Fourier-transform infrared spectroscopy analysis demonstrated an 87% degradation rate of DBDE for the ABS-DBDE system and 91% for the PC-DBDE system following an 1800 kGy irradiation dose. Thermal analysis using Differential Scanning Calorimetry revealed the presence of crosslinking in ABS and a minor reduction in the glass transition temperature of PC after EB processing. Polymers exhibited thermal stability after photolysis, as indicated by thermogravimetric analysis. In summary, EB irradiation had no impact on the overall thermal properties of both polymers. High-resolution mass spectrometry analysis has confirmed the debromination of both ABS-DBDE and PC-DBDE systems. Therefore, the results obtained are promising and could offer an alternative approach for removing bromine and other additives from plastic E-waste.

## 1. Introduction

New policies promoting the circular economy of Waste Electrical and Electronic Equipment (WEEE) aim to foster recycling innovations [1,2]. Current plastic waste incineration practices generate significant pollution and health impacts, as well as substantial economic and energy losses over time [3,4]. Polybrominated diphenyl ethers (PBDEs) are a group of halogenated biphenyls containing bromine atoms that have been used extensively as Brominated Flame Retardants (BFRs) in a variety of end products, including plastics, construction, textiles, electronics, and furniture materials [5,6]. PBDEs were historically used in various forms since the 1960s and continued to be used for nearly five decades before most congeners were banned [7].

PBDEs are among the most significant environmental pollutants due to their resistance to degradation. These lipophilic molecules accumulate in the fatty tissues of living organisms and cause various pathologies [6]. The primary methods of human PBDE exposure involve ingesting and inhaling dust [8]. PBDEs have been detected in human samples such as blood, placental tissue, and breast milk [9], and their half-life in the human body is estimated to range from 1 to 12 years [10,11]. These chemicals act as endocrine disruptors, having an impact on neurological development in humans [12,13], various male reproductive functions [14], and the incidence of abnormal genital development [15,16].

**Figure 1:**
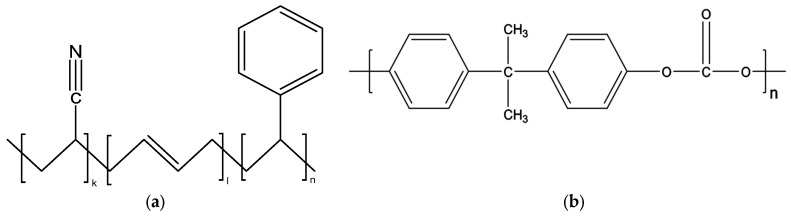
Chemical structures of the investigated polymers (**a**) Acrylonitrile-butadiene-styrene (ABS), (**b**) Polycarbonate (PC).

According to surveys, the highest production of PBDEs was recorded in 2003, reaching almost 90,000 tons per year [16]. Since then, various measures have been taken to limit the use of PBDEs around the world, until they were finally banned in Europe by the ROHS II Directive (2011/65/EU). In 2017, a new law was passed limiting their use to the aero-space and automotive industries [17]. Thousands of wastes contaminated with these toxic molecules are currently present in the environment. The PBDE-containing polymers most often found in WEEE are acrylonitrile butadiene styrene (ABS) and polycarbonate (PC) and its derivatives [18,19]. Their corresponding chemical structures are presented in Figure 1. To date, various biological, chemical, and physical treatments have been studied and evaluated to eliminate PBDEs [20]; however, obtaining industrial scale still remains a real economic challenge.

In recent years, electron beam (EB) processing has become a promising tool in both basic and applied sciences. The plastics, automotive, electrical wire, and cable industries are increasingly using EB processing to improve articles’ quality by enhancing the long-term thermal and mechanical properties of materials [21]. At present, EB processing is widely employed in industry. The technology’s time and energy savings, along with its efficiency, have accelerated commercialization in a cost-effective manner. Moreover, this solvent-free technique is acknowledged as eco-friendly, as it significantly decreases emissions of volatile components [22]. It has also demonstrated efficacy in decontaminating packaging materials for food and pharmaceuticals. This technology has the ability to sterilize medical devices, cosmetics, and pharmaceutical products through its dissociation of high-energy radiation [23]. Furthermore, EB technology has various uses in aerospace and environmental applications, and is being researched in various fields, including those that seek to provide structural parts for automotive panels, electro-optical devices and healthcare products [24].

The possibility of 4-Bromodiphenylether, a BFR, being degraded by EB irradiation at various pH levels in an aqueous treatment has been demonstrated. Laser Flash Photolysis analysis observed a degradation efficiency of 99.8% at pH = 10 with a dose of 14 kGy [25]. Zhao et al. investigated both flooded acetone/water as well as non-flooded soils that were contaminated with Decabromodiphenylether (DBDE) [26]. The optimal results for debromination were observed in the flooded soils with irradiation doses up to 50 kGy.

**Figure 2:**
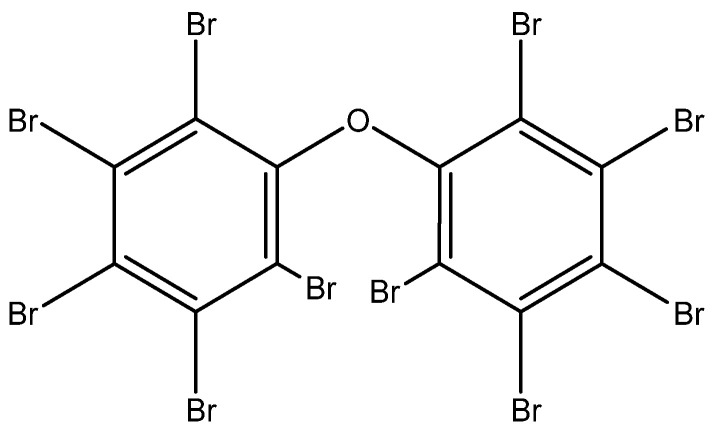
Chemical structure of Decabromodiphenylether (DBDE).

Two model systems were studied in this report. They consisted of 10% of DBDE by weight, dispersed in industrial grade linear polymers (ABS and PC) at a weight percentage of 90%. The systems were subjected to EB irradiation to investigate debromination effects. DBDE was chosen for this study because it is one of the most toxic and persistent BFR compounds in WEEE, which is commonly used as a plastic additive (refer to Figure 2).

Some thermal properties of ABS and PC samples after EB irradiation were compared with the pristine polymers to assess the feasibility of reusing the decontaminated material.

## 2. Results and Discussions

The debromination of thin films of ABS-DBDE and PC-DBDE was examined by Fourier-transform infraRed (FTIR) spectroscopy and high-resolution mass spectroscopy post exposure to EB irradiation. The blend consisting of 90% ABS and 10% DBDE is named ABS-DBDE, while the blend consisting of 90% PC and 10% DBDE is named PC-DBDE. The corresponding samples subjected to irradiation are labeled as ABS-DBDE x kGy and PC-DBDE x kGy, where x denotes the specific dosage value.

### 2.1. Infrared Spectroscopy Studies of DBDE Degradation in PC and ABS

#### 2.1.1. Polycarbonate-Decabromodiphenylether

**Figure 3:**
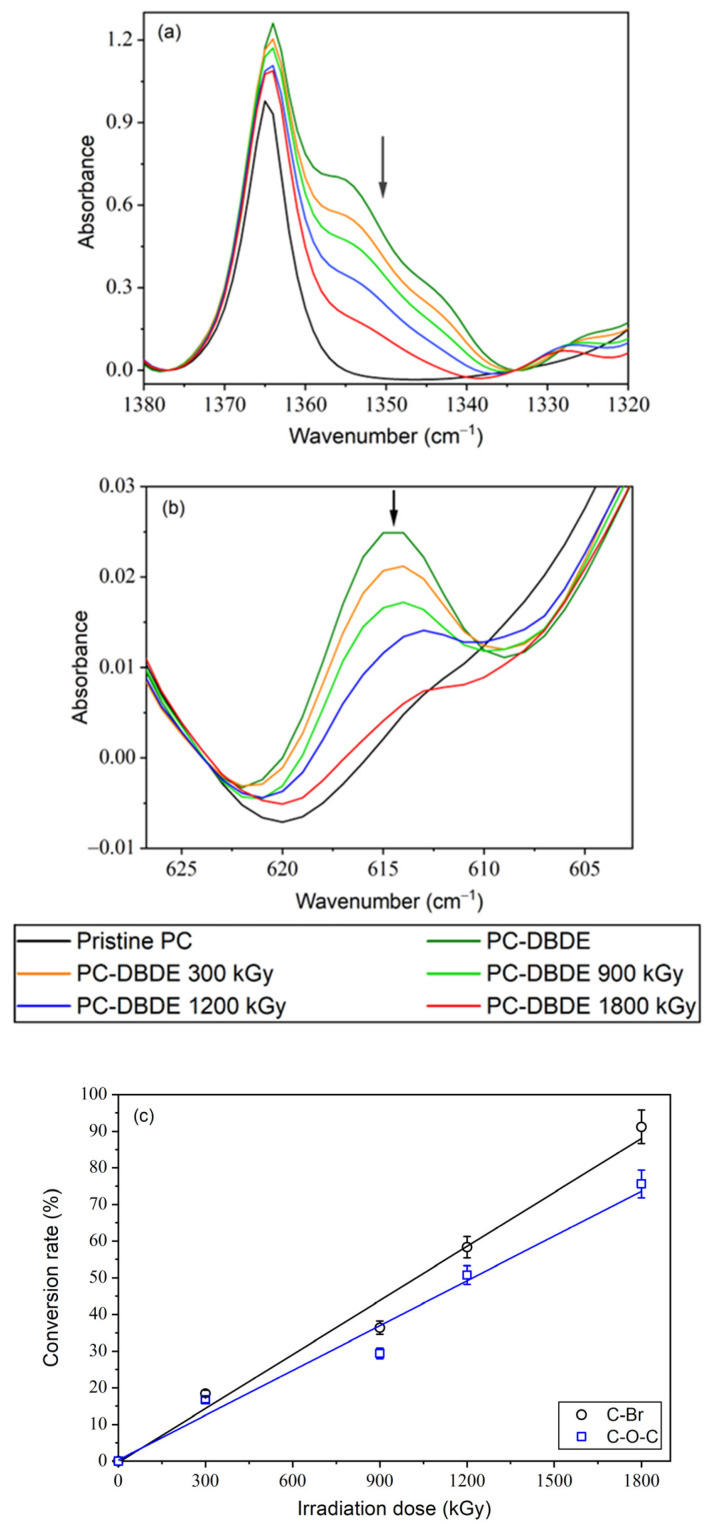
FTIR spectra of PC-DBDE as a function of EB dose: (**a**) evolution of the C-O-C ether band of DBDE between 1335 and 1380 cm^−1^; (**b**) evolution of the aromatic C-Br band of DBDE between 605 and 625 cm^−1^; (**c**) DBDE conversion rates for C-Br and C-O-C bands. The arrows were added to emphasize the decrease in the investigated bands and the lines represent guides for the eyes.

FTIR spectra for pristine PC, PC-DBDE, and EB-irradiated PC-DBDE at varying doses are displayed in Figure 3. Two vibrational bands were utilized to monitor the degradation of DBDE. Specifically, Figure 3a illustrates the progression of the band found between 1335 and 1380 cm^−1^, which corresponds to the C-O-C ether function of DBDE. This band is accountable for the strength of the molecule and is the most dominant band within the FTIR spectrum of DBDE. Figure 3b illustrates the gradual decline of a weak band, with a maximum around 615 cm^−1^, which corresponds to the aromatic C-Br band of DBDE [27].

Based on the findings presented in Figure 3, increasing the EB doses causes a significant decline in the intensity of the specified vibrational bands. This indicates the degradation of the DBDE molecule and the elimination of bromine from the polymer film.

#### 2.1.2. Acrylonitrile Butadiene Styrene-Decabromodiphenylether

**Figure 4:**
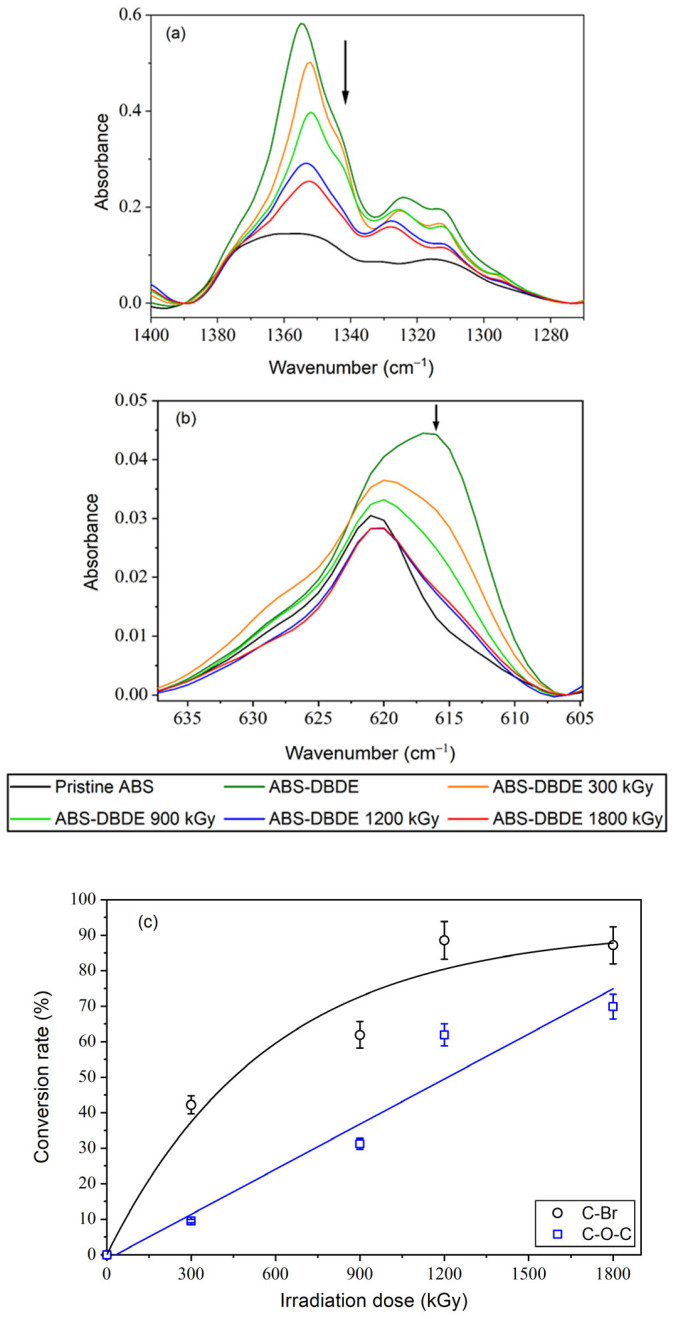
FTIR spectra of ABS-DBDE as a function of EB dose: (**a**) evolution of the C-O-C ether band vibration of DBDE between 1280 and 1390 cm^−1^; (**b**) evolution of the aromatic C-Br band vibration of DBDE between 605 and 635 cm^−1^; (**c**) DBDE conversion rates for C-Br and C-O-C bands. The arrows were added to emphasize the decrease in the investigated bands and the lines represent guides for the eyes.

Figure 4 displays the FTIR spectra of ABS-, ABS-DBDE-, and EB-irradiated ABS-DBDE as the dose varies. Regarding PC-DBDE, the intensity of the C-O-C ether band of DBDE in the range of 1280–1390 cm^−1^ decreased (as shown in Figure 4a) with an increase in EB dose. Furthermore, the intensity of the C-Br band of DBDE between 605 and 635 cm^−1^ also decreased as the EB dose increased. This indicates a near-complete elimination of alkyl- and aryl-bromides from the polymer.

The DBDE conversion rates (expressed in %) were determined through the calculation of (H_0_ − H_D_)/H_0_ × 100, with reference to the absorbance of the band peaks that correspond to 615 cm^−1^ (C-Br band vibration) and 1350 cm^−1^ (C-O-C band vibration) for DBDE. In this study, H_0_ represents the non-irradiated PC/ABS-DBDE films while H_D_ corresponds to the irradiated ones. The latter is presented as a function of the radiation dose D. For PC-DBDE, the conversion rate of DBDE displays a linear dependence on the radiation dose for both the evolution of the C-O-C band and the C-Br vibration (refer to Figure 3c). There is a linear correlation between the development of the C-O-C band in ABS-DBDE, while the conversion rate linked to the C-Br band appears to exhibit an exponential tendency (refer to Figure 4c).

**Figure 5:**
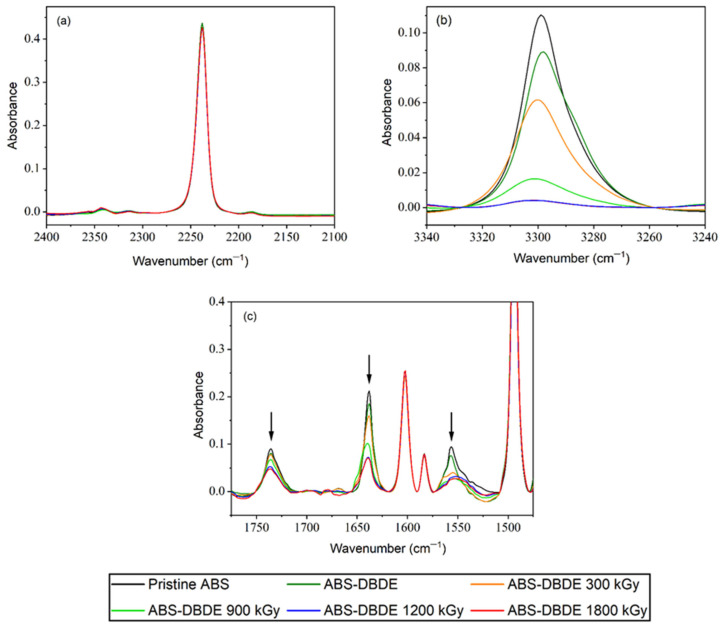
FTIR spectra of ABS-DBDE as a function of EB dose: (**a**) C≡N band vibration of ABS around 2250 cm^−1^; (**b**) N-H band vibrations of polymer additives between 3240 and 3340 cm^−1^; (**c**) N-H band vibrations of polymer additives between 1475 and 1775 cm^−1^. The arrows were added to emphasize the decrease in the investigated bands.

Figure 5a indicates that the C≡N band of the acrylonitrile group, which is situated around 2250 cm^−1^, remained stable during EB processing. This suggests that the acrylonitrile part of the triblock copolymer shows some resistance to irradiation. However, Figure 5b,c exhibits a reduction in the intensity of specific bands, including 3300, 1735, 1640, and 1555 cm^−1^, possibly related to vibrations of nitrogen-containing groups. The ABS and PC materials contain polymeric additives carrying nitrogen groups, including chemical blowing agents, lubricants, and antistatic agents. For ABS and PC, azoimides such as azodicarbonamide serve as blowing agents and produce IR bands around 3300, 1730, and 1640 cm^−1^ [28]. The location of similar bands is evident in Figure 5b,c. Additionally, the long-chain fatty acid amides appear as slipping agents that contribute to the IR spectrum, and exhibited bands localized around 3300, 1640, and 1555 cm^−1^ [29]. Furthermore, antistatic agents such as ammonium salts or ethoxylated amines also contribute to vibrational bands associated with nitrogen groups. It can be concluded that these polymer additives tend to disappear as a result of EB irradiation. Therefore, the decontaminated recycled polymers require re-additivation, which is a common procedure for producing new products.

### 2.2. Detection of BFR with HR-L2MS Mass Spectrometry

**Figure 6:**
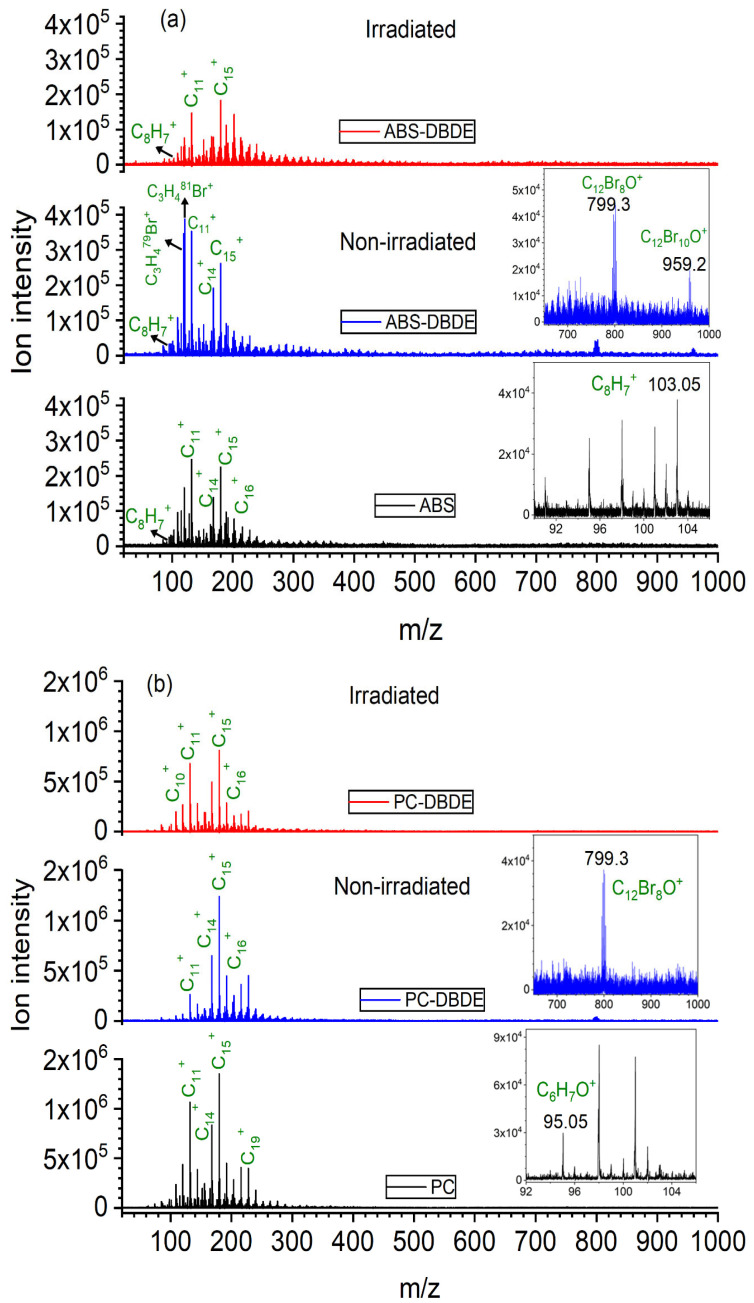
Positive ion mode mass spectra: (**a**) Comparison of HR-L2MS spectra of pure ABS (black color), non-irradiated (blue color) ABS-DBDE, and irradiated ABS-DBDE 1800 kGy (red color); (**b**) Comparison of HR-L2MS spectra of pure PC (black color), non-irradiated (blue color) PC-DBDE, and irradiated (red color) PC-DBDE 1800 kGy.

Figure 6 presents the mass spectra acquired in positive mode for pure ABS and PC, and their mixtures with DBDE before and after exposure to EB irradiation. The recorded spectra display strong peaks, indicating C_n_^+^ carbon clusters are present in all samples. Furthermore, ABS and PC mass spectra reveal peaks related to protonated phenol (C_6_H_7_O^+^, 95 *m*/*z*) and deprotonated styrene (C_8_H_7_^+^, 103 *m*/*z*), respectively. All of these compounds originate from the fragmentation of carbon-based materials, especially polymers. Additionally, several mass peak series, centered at 119 *m*/*z*, 121 *m*/*z*, 799 *m*/*z*, and 959 *m*/*z*, were identified in the non-irradiated ABS/PC-DBDE sample. These peaks correspond to specific isotopic distributions of bromine compounds, including C_3_H_4_Br^+^, octabromodiphenylether (C_12_Br_8_O^+^), and decabromodiphenylether (C_12_Br_10_O^+^). In contrast to the previous species, these peaks disappear from the spectra following EB irradiation, suggesting a debromination effect.

**Figure 7:**
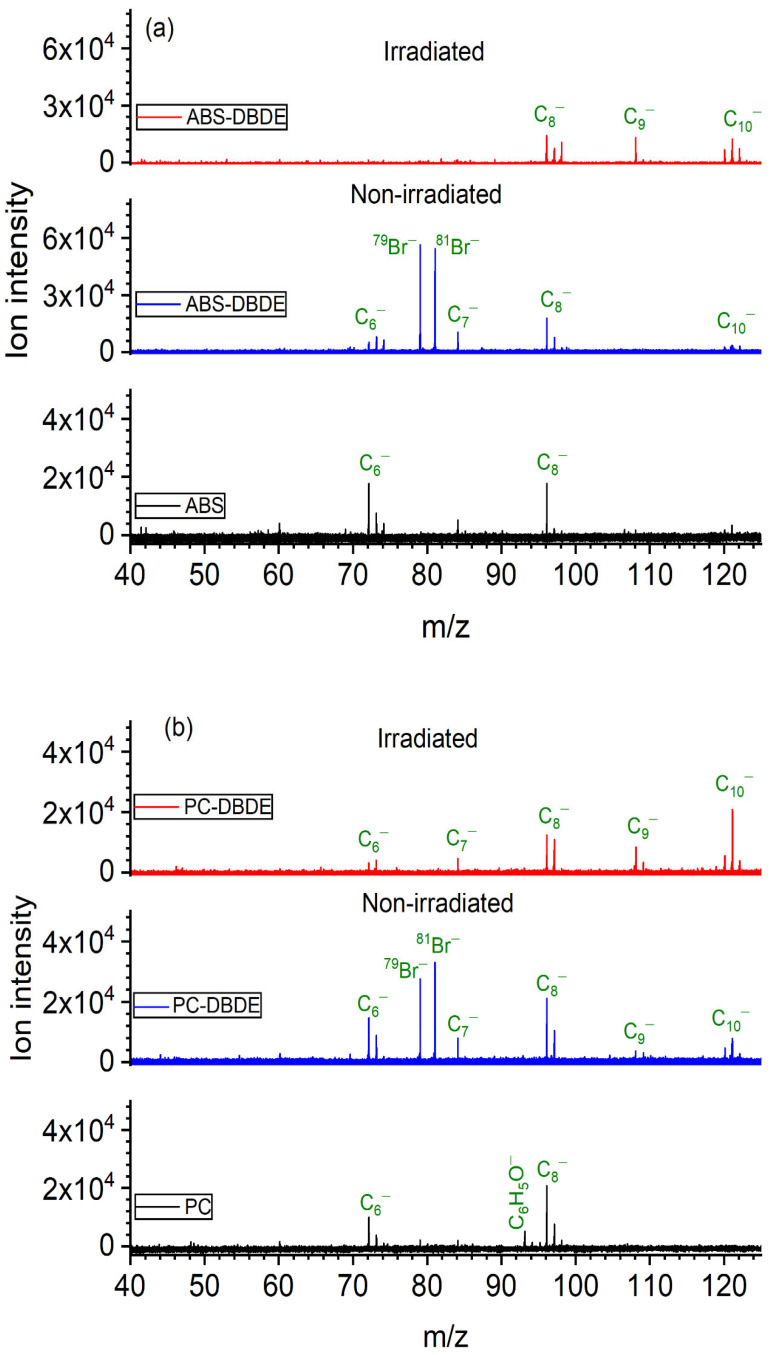
Negative ion mode mass spectra: (**a**) Comparison of HR-L2MS spectra of pure ABS (black color), non-irradiated (blue color) ABS-DBDE, and irradiated ABS-DBDE 1800 kGy (red color); (**b**) Comparison of HR-L2MS spectra of pure PC (black color), non-irradiated (blue color) PC-DBDE, and irradiated (red color) PC-DBDE 1800 kGy.

The hypothesis is supported by the negative mode spectra presented in Figure 7. Although these spectra are less complex than their counterparts, they still exhibit peaks at 79 and 81 *m*/*z*, which suggests the bromide ion is exclusively present in the mass spectra of PC/ABS-DBDE. The significant reduction in all bromine peaks within the polymer/DBDE blend following exposure to EB irradiation suggests that the elimination of the brominated species occurred in the film, rather than just fragmenting during the treatment.

**Figure 8:**
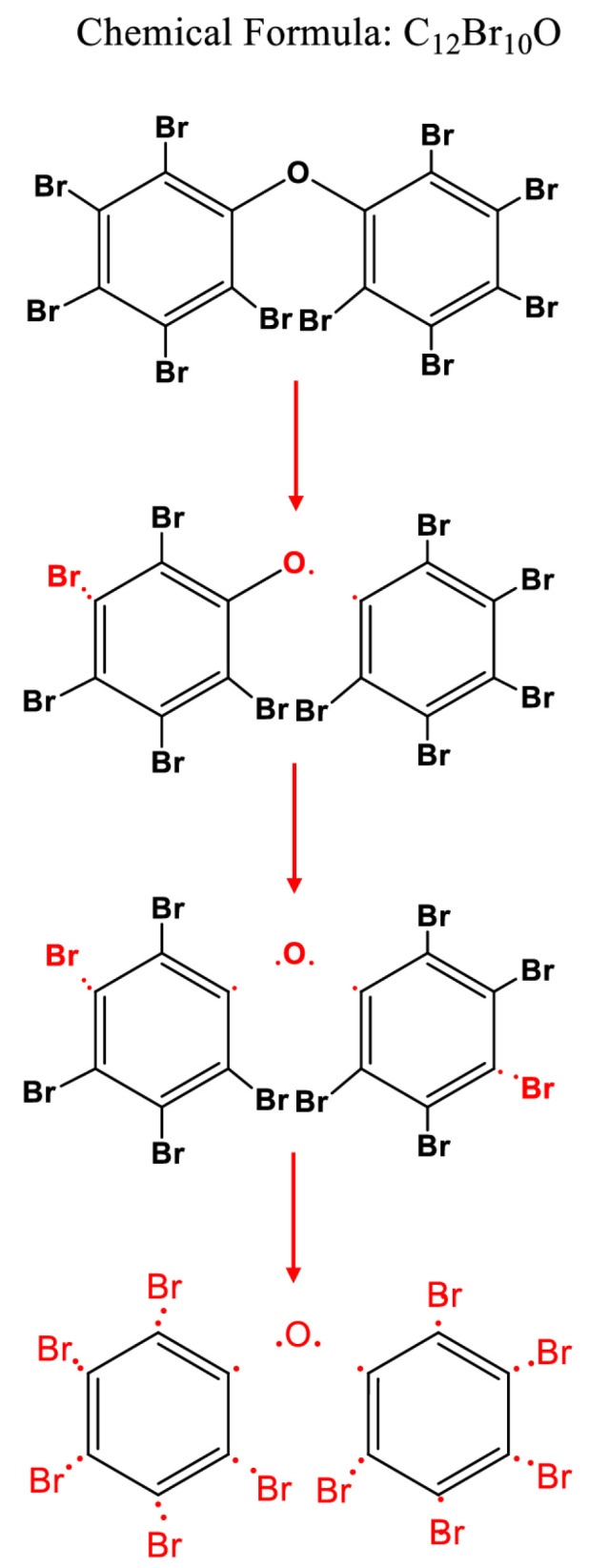
Schematic diagram of the degradation of DBDE.

The FTIR and HR-L2MS results suggest the degradation of DBDE occurs through simultaneous scission of C-O-C and C-Br bonds. Figure 8 illustrates the successive stages of DBDE degradation induced by EB irradiation.

### 2.3. Thermal Properties of PC and ABS

#### 2.3.1. Differential Scanning Calorimetry

**Figure 9:**
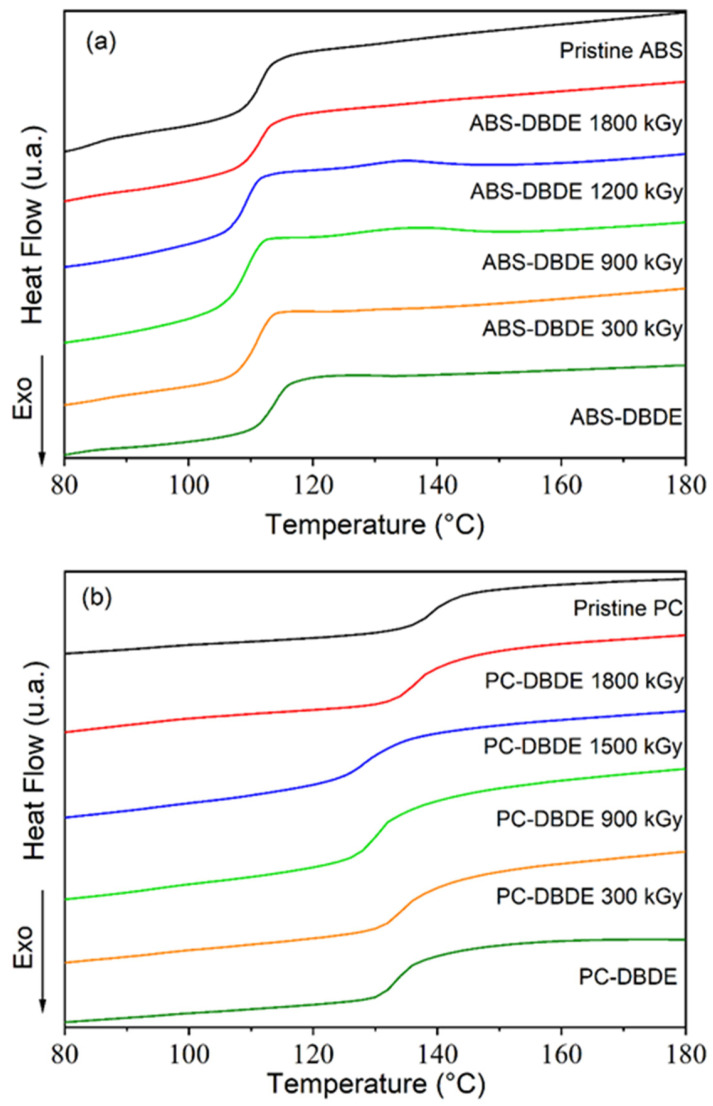
DSC thermograms of: (**a**) pristine ABS, ABS-DBDE and EB-irradiated ABS-DBDE; (**b**) pristine PC, PC-DBDE, and EB-irradiated PC-DBDE.

A DSC analysis was conducted to examine the thermal properties of ABS-DBDE and PC-DBDE before and after irradiation. Figure 9a displays the thermograms acquired for the unaltered ABS, ABS-DBDE, and EB-irradiated ABS-DBDE at varying doses. On the other hand, Figure 9b depicts the corresponding scenario for PC. The detection of a single glass transition (T_g_) in each sample indicates the lack of phase separation effects. Interestingly, the addition of DBDE to virgin ABS causes a small change in T_g_ (Figure 9a), which is unlike the case of PC-DBDE where the incorporation of DBDE results in a significant change in T_g_ (Figure 9b).

The ABS sample results (Figure 9a) reveal a 3 °C increase in T_g_ after exposure to 1800 kGy irradiation, a rise attributable to a weak cross-linking effect. Subsequently, this slightly improves the thermal stability of the original ABS [30]. Additionally, a weak glass transition was observed at approximately −42 °C (not depicted), corresponding to the butadiene fraction of ABS [31].

Figure 9b illustrates that the degradation of DBDE induced by irradiation in PC-DBDE results in a slight reduction in T_g_. Increasing the EB dose to an increase in the formation of lower-molecular-weight molecules (that is initiated by C-O-C and C-Br cleavages) triggers a plasticizing effect that explains the decrease in T_g_. A reduction of 8 °C was observed from PC-DBDE to PC-DBDE 1500 kGy. Meanwhile, T_g_ increases beyond 1500 kGy, approaching the value of pristine PC for PC-DBDE 1800 kGy. One hypothesis that accounts for this phenomenon is the evaporation of low-molecular-weight degraded species, which results in a diminished plasticizing impact.

#### 2.3.2. Thermogravimetric Analysis

**Figure 10:**
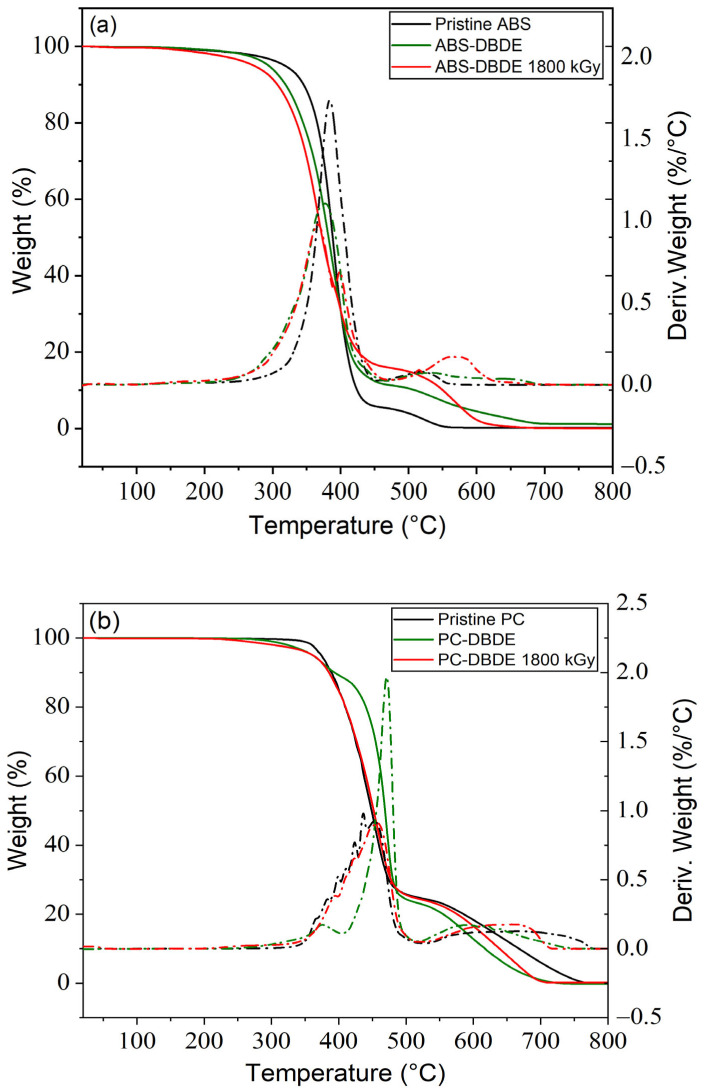
Weight losses (continuous lines) and their derivatives (dashed lines) obtained by TGA for: (**a**) pristine ABS, ABS-DBDE and EB-irradiated ABS-DBDE; (**b**) pristine PC, PC-DBDE and EB-irradiated PC-DBDE.

Figure 10a displays the outcomes of the thermogravimetric analysis of pristine ABS, ABS-DBDE, and ABS-DBDE 1800 kGy. The decomposition profiles show that thermal degradation occurs in two phases for all samples. In the case of pristine ABS, thermal degradation (resulting in a mass loss of 5 wt%) begins at roughly 320 °C and is followed by two degradation stages at around 390 °C and 500 °C, respectively.

The addition of DBDE to ABS results in decreased thermal resistivity of ABS-DBDE in comparison to pure ABS. Indeed, degradation initiates at 5% mass loss around 295 °C, followed by two subsequent degradation stages at temperatures similar to those shown for pure ABS. A difference of roughly 10 wt% in mass loss is observed for the second degradation step when comparing results from ABS with ABS-DBDE, indicating that thermal degradation of DBDE primarily occurs during this step.

Figure 10a displays that ABS-DBDE 1800 kGy experiences thermal degradation between 400 and 600 °C, which results from the degradation of partially formed cross-linked ABS-chains while undergoing EB irradiation [32,33]. Tiganis et al. [33] previously studied the thermal decomposition of ABS and used various analytical techniques to observe surface deterioration through chain scission and cross-linking in the polybutadiene phase of ABS. According to Yang et al. [34], thermal degradation effects can originate in various factors, such as the sample weight ranging from 5 to 60 mg, heating rates between 10 and 40 °C per minute, purge gases like N_2_/Air [35], a purge gas rate from 25 to 80 mL per minute, and molecular weight, as well as ratios of acrylonitrile, butadiene, and styrene in the sample itself. Multiple factors impact the degradation of ABS, yet a straightforward comparison between the literature findings and the presented results cannot be made due to variable instrumental conditions.

Figure 10b displays TGA curves for pristine PC, PC-DBDE, and PC-DBDE 1800 kGy. In contrast to the ABS case, the degradation curve of unaltered PC displays a diminished thermal stability in comparison to PC-DBDE, at least during the initial stage.

The results show that PC-DBDE 1800 kGy and pristine PC undergo similar degradation curves, which could be a sign of successful DBDE degradation.

## 3. Experimental Section

### 3.1. Materials

DBDE as a BFR was purchased from Greenchemical S.p.a. (Desio, Italy). ABS was obtained from Chi Mei Corporation (Tainan, Taiwan) via AMP Polymix (Horbourg-Wihr, France), and PC was purchased from Covestro AG (Leverkusen, Germany). Mixtures of each polymer were combined with 10 xt% of DBDE using a micro-extrusion machine Micro 15HT from Xplore Instruments BV (Sittard, Netherlands) that was equipped with a twin-screw extruder. Small pellets were produced under conditions of 185 °C, 5 bars of pressure, and a screw speed of 150 tr/min. The average diameter size of all pellets ranged between 1 and 3 mm.

### 3.2. Electron-Beam Processing

EB processing was conducted using a COMET/EBLab machine from Comet located in Flamatt, Switzerland, with an operational high voltage of 180 kV. To achieve complete penetration of the samples through EB irradiation, thin films were produced from ABS, ABS-DBDE, PC, and PC-DBDE pellets with a thickness ranging between 30 and 50 μm. This allowed for the transformation of the pellets into the desired thin films. A laboratory molding press, the Servitec Polystat 200T from Servitec Maschinenservice GmbH in Berlin, Germany, was utilized, and gradually applied 90 bars over 4 min at 230 °C. The thin films obtained were placed on a tray positioned on a conveyor belt, and operated under a nitrogen atmosphere. To achieve a dose of 300 kGy for one pass, a conveyor speed of 3 m/min and a beam intensity of 8.161 mA were chosen. Higher dosage values of 600, 900, 1200, 1500, and 1800 kGy were obtained through multiple passes of 300 kGy.

### 3.3. Fourier Transform Infrared Spectroscopy

The degradation of DBDE induced by EB irradiation was observed through the debromination of ABS-DBDE and PC-DBDE using FTIR to analyze changes in the molecular structure. The analysis was carried out using a PerkinElmer Frontier spectrometer (Perkin Elmer, Waltham, MA, USA). The polymer films were recorded in transmission mode within a spectral range of 500–4000 cm^−1^ at ambient temperature. The number of accumulated scans was 16, with a spectral resolution of 4 cm^−1^. The collected spectra were corrected for baseline and normalized to a predetermined wavenumber of the polymer.

### 3.4. Differential Scanning Calorimetry

The glass transition temperatures of both the pristine and EB irradiated samples were obtained by utilizing the DSC Q2000 instrument from TA Instruments (New Castle, DE, USA). To prepare the samples, 9 ± 3 mg of polymers were added to an aluminum crucible. The received heat flow data were standardized to the weight of the samples and a heating and cooling rate of 10 °C/min was maintained within the temperature range of −70–+200 °C under a nitrogen flow. The initial step of the program cooled the sample and carried out three cycles of heating and cooling to account for any thermal events that might be linked to the sample’s preparation history. The thermograms analyzed in this study were obtained during the second heating cycle. To ensure the reproducibility of the results, at least three experiments were conducted using identical samples with the same composition and independent preparation. The glass transition temperatures were obtained through the calculation of the midpoint within the transition range of the thermograms.

### 3.5. Thermogravimetric Analysis

A thermogravimetric analysis was conducted of the specified samples using a Pyris 1 instrument from PerkinElmer (Waltham, MA, USA) with a 1 µg mass resolution. Samples with an average mass of 8 mg in platinum pans were prepared and processed in a nitrogen environment with a heating ramp of 10 °C/min. Samples with an average mass of 8 mg in platinum pans were prepared and processed in a nitrogen environment with a heating ramp of 10 °C/min. Polymer processing occurred at temperatures ranging from 100 to 900 °C.

### 3.6. High-Resolution Two-Step Laser Mass Spectrometry (HR-L2MS) Analysis

The polymeric films were analyzed using High-Resolution Two-step Laser Mass Spectrometry (HR-L2MS), following a detailed experimental setup described elsewhere [36]. To summarize, the film was placed on a copper plate inside a high-vacuum chamber and exposed to normal surface irradiation with nanosecond pulses of a frequency-doubled Nd: YAG laser (Quantel Brilliant EaZy, wavelength: 532 nm, pulse duration: 4 ns, repetition rate: 10 Hz, fluence: 1.5 J·cm^−1^). Neutrals were emitted from a plume that extended normally to the surface of the film within a vacuum. These neutrals were ionized through multi-photon processes using 10 ns pulses generated by a frequency-quadrupled Nd: YAG laser beam (Quantel Q-smart 850, wavelength: 266 nm, pulse duration: 5 ns, repetition rate: 10 Hz, fluence: 7.5 × 10^−2^ J·cm^−2^), which was focused perpendicularly on the direction of the plume’s propagation. The ion packets were directed to an octupole ion trap where they collided with helium atoms injected from a fast solenoid valve, resulting in thermalization. This process produced high spectral mass resolution (m/Δm~15,000). The ions were analyzed using a time-of-flight mass spectrometer equipped with a reflectron (ToF-MS) to detect cations or anions in either positive or negative mode. The mass spectra of each film corresponded to the accumulation of 300 laser shots. During each acquisition, the film shifted along two axes by means of Attocube’s piezoelectric nanopositioners, maintaining a minimum shift of 100 µm to guarantee that the next laser shot targeted a new spot on the film.

## 4. Conclusions

Model systems of industrial polymers (ABS and PC) containing 10 wt% DBDE were selected for this study due to their prevalence in WEEE. Thin polymer films (30–50 µm) were used by this study because the penetration of electrons into the polymeric material is restricted by laboratory equipment. The rate of degradation was assessed by FTIR spectroscopy following EB irradiation. Results showed an 87% degradation rate for the ABS-DBDE system and 91% for the PC-DBDE system, both after exposure to an irradiation dose of 1800 kGy. The total bromine content was measured using high-resolution mass spectrometry, both before and after EB irradiation. This study found that after irradiation, the bromine content was almost non-existent, meeting the legal standards for recycling and reusing decontaminated polymers. The thermal analysis, conducted through DSC, identified a minor crosslinking effect on ABS and a slight reduction in the glass transition temperature for PC following EB processing.

## Data Availability

The data presented in this study are available on request from the corresponding author.
